# Factors affecting home gardens ownership, diversity and structure: a case study from Benin

**DOI:** 10.1186/s13002-015-0041-3

**Published:** 2015-07-09

**Authors:** Rodrigue Castro Gbedomon, Adandé Belarmain Fandohan, Valère Kolawolé Salako, Alix Franck Rodrigue Idohou, Romain Glèlè Kakaї, Achille Ephrem Assogbadjo

**Affiliations:** Laboratory of Biomathematics and Forest Estimations, Faculty of Agronomic Sciences, University of Abomey-Calavi, 04 BP 1525 Cotonou, Benin; Laboratory of Applied Ecology, Faculty of Agronomic Sciences, University of Abomey-Calavi, 01BP 526 Cotonou, Benin; Forestry, Agroforestry and Biogeography Unit, University of Agriculture of Kétou, BP 43 Kétou, Benin; Abteilung für Biometrie und Umweltsystemanalyse, Albert-Ludwigs-Universität Freiburg, Tennebacher Str. 4, 79085 Freiburg, Germany

**Keywords:** Home gardens, Ownership, Plant diversity, Socio-economic, Conservation, West Africa

## Abstract

**Background:**

Home gardens (HGs) provide perspectives for conservation of plant genetic resources while contributing to improving livelihoods. However, knowledge of local factors shaping their ownership, plant diversity (PD) and structure is still limited especially in West-Africa, where food insecurity is acute. This is critical to ensure effective mainstreaming of HGs into future biodiversity conservation and food production policies.

**Methods:**

Socio-economic and PD data were obtained from individual interviews (*n* = 470) and gardens inventories (*n* = 235) spanning humid, sub-humid and semi-arid zones of Benin. Generalised Linear Models, Hierarchical Cluster Analysis, Principal Component Analysis and Simple Correspondence Analysis were performed to examine socio-economic characteristics (age, gender, education level and main economic activity) affecting HGs ownership, and their effect coupled with intrinsic HGs characteristics (size, age) on PD and structure within HGs, across contrasting bio-geographical regions.

**Results:**

HG ownership was significantly dependent upon a complex relationship between age, gender and education level of the farmers. The probability to own HG increased with age with an early involvement in home gardening for women. Similarly, with increasing age, it was more likely to find a male owner than a female owner among the uneducated informants and those of primary school. Inversely, it was more likely to find female owner than a male owner among secondary school level or more.

PD increased with increasing owner age and size of the HG. Larger and more diversified HGs were found in sub-humid and semi-arid zones while smaller and less diversified HGs were encountered in the humid zone. HGs were multi-layered. Based on the prevailing plant groups, three categories of HG were distinguished: Herb based gardens, Herb and Shrub/Trees based gardens, and Palm and Liana based gardens. Their prevalence was dependent upon bio-geographical zones and HG owner socio-economic characteristics, with herbs based HGs being mainly associated to women.

**Conclusion:**

Results suggest effects of complex interactions between socio-economic factors on HG ownership, and influence of these effects combined with intrinsic characteristics of HGs on PD. The early involvement of women in home gardening and their particular interest in herbs and shrubs are important assets for future conservation strategies based on HG and food production. Interventions are required to interfere with declining PD in HG across generations to accommodate multiple ecosystem service benefits.

## Background

Feeding a dramatically growing population while conserving natural resources is one of the greatest contemporary challenges decision makers face. Concerns are even greater in areas where people rely on these natural resources for their daily needs. Interestingly, there is a growing interest in integrating native Plant Genetic Resources (PGR) into formal farming systems, as a critical step towards achieving the Millennium Development Goal 1 “*combating extreme poverty and hunger*” [1]. Similarly, with regard to land use dynamics, this step is perceived as a more realistic approach to conservation [2–4]. Conservation of PGR is vital not only because of their prominent role in food and agriculture [5] but also for the adaptation of certain cultivars to predicted changes in the climate, diseases and pests in the future [5–7]. The growing interest on agroforestry systems as the future of PGR conservation can be explained by the optimal ecological conditions and the protection provided to plants in these traditional farming systems [6]. Among these traditional farming systems, Home gardens (HGs) have attracted attention in the last decades.

HGs are a cultivated space, generally adjacent to a household or slightly further away but still easily accessible [8]. The number of studies focusing on HG has recently increased worldwide with special focus on their roles in improving rural households’ livelihoods while conserving biodiversity. For instance, HGs have been reported to support food system [9–12], mitigate economic hardship and provide additional income for households [13–16]. In addition, they enhance the empowerment and social position of women [17–19]. HGs have been also reported effective in agro-biodiversity conservation [20–22], ecosystem services provision [23] and culture preservation [17, 24, 25].

However, information on African HGs (as compared to their tropical Latin American counter parts) is relatively poor. They have been for long time been neglected by academic research and development policies [26–28]. Existing literature on African HG focuses mainly on their contribution to global household food and nutrition [29–36], uses and traditional knowledge associated to them [37] as well as their plant diversity and potential contribution to biodiversity conservation [22, 38–41]. However, with the current socio-cultural dynamics in Africa i.e. erosion of traditional knowledge of plant and associated uses [42], westernization of production and consumption systems [43, 44], increasing use of improved crops [45, 46] and rapid population growth [47], traditional HGs are threatened [11]. Thus, their potential for biodiversity conservation is questionable. In this line, how local factors shape ownership of HG and how these factors coupled with garden features determine plant diversity and structure in HG are important questions to be addressed. Investigating these aspects of HGs is a prerequisite to ensure effective mainstreaming of HGs into future conservation and production policies. Therefore, this study explored the socio-economic factors influencing HG ownership and how these factors combined with intrinsic characteristics of HGs, shape their plant diversity and structure across three bio-geographical zones in the republic of Benin.

Traditionally, women and old men used to cultivate fields close to their village, while the younger men often set their fields a bit further from their village [48]. With this traditional specialization in land use, we expected home gardens to be mostly owned by women and old persons. In addition, as western school education influence lifestyle including farm production purposes [38], we expected home gardens to be owned mainly by uneducated people. As home gardening is an agricultural practice, we expected HGs to be owned mainly by people with on-farm activities. We also expect to find significant interactions among these factors. Furthermore, based on previously reported influence of socio-economic conditions of HG owners [38, 49–51] and intrinsic characteristics [51–53] on plants maintained in HGs in Latina America, we predicted that plant diversity in HG would also increase with age and size of the HG, both increasing with the age of the owner.

In this study, plant diversity refers to the diversity of cultivars, landraces, ecotypes, wild relatives and wild plants, deliberately maintained in HGs. It excludes weedy species which are spontaneous vegetation, removed by the owner as often as possible. Furthermore, by shaping plant diversity in HG, ecological conditions [50], socio-economic factors , preferences of HG owners but also market opportunities [40, 41] control the structure of the HGs in maintaining an assemblage of herbs, vines, shrubs and trees. Generally, the HGs structure refers to the composition, including spatial arrangement of the woody component, vertical stratification and temporal arrangement of the different components [54]. Here, we focused on composition (with plant richness and diversity of CWR as estimates) and vertical stratification (with prevailing plant group as estimate) and did not address the temporal arrangement of the HGs. Like many tropical HGs [54], we predicted HGs in Benin to be diversely composed and multilayered. We further predicted that the structure of the HGs would be dependent upon bio-geographical zones and socio-economic conditions of the HGs owners.

## Methods

### Study area

The study was conducted in Benin, a West African country of 115762 km^2^ [55], located between 6°25’N-12°30’ and 0°45’E - 4°E and characterized by three contrasting bio-geographical zones [56] ranging (based on days of growing season) from humid to semi-arid zones [57].

The country’s native vegetation has suffered severe degradation as a result of various intense anthropogenic activities. In the southern part i.e. the humid zone, where the population density is high, the vegetation is composed of fallows and small forest patches of less than 5 ha [58]. The transition zone i.e. the sub-humid zone is characterized by mosaics of woodlands while the vegetation in the semi-arid zone (commonly Sudanian zone) consists of savannas and gallery forests with trees and shrubs slightly covering the ground.

The resident population of about 9 983 884 inhabitants is unequally distributed [59], with 60 % of the population concentrated in 20 % of the territory [60]. The population is mainly young (more than40 % is under 15 years old ) and slightly female-biased (51.2 %) [59]. Thirty three percent of the population has at least basic education (Primary school or alphabetization in local languages) while the remaining part of the population can neither read nor write [60]. The local economy is agriculture-based [59]. More than half the population (53.9 %) lives with less than USD 1.00 per day [61] and 35 % of the populations is in food insecurity [62]. Agriculture sector employs more than 70 % of the active population in rural areas [63]. Important cash crops are: cotton in the semi-arid zone, cashew in the sub-humid zone and palm oil in the humid zone. Important food crops are: sorghum and maize (semi-arid zone), yam and maize (sub-humid zone) and, cassava and maize (humid zone). The average size of farmland per farmer is 1.7 ha and 34 % of the 550,000 farms inventoried in Benin cover less than 1 ha [62]. The sub--humid zone where population density is relatively lower is experiencing increasing “agricultural colonisation” by farmers from the semi-arid and humid zones. Generally, regardless of ethnic groups, women have no or limited access to land [62]. Women use to work with their husband and/or hire small pieces of land for their activities; and as tenant, they are not allowed to establish perennial crops (i.e., fruit trees), but rather short-cycle crops.

In Benin, 55 individual languages have been listed and could be grouped into three major socio-linguistic groups namely Kwa, Gur and Yoruboїd [60]. The socio-linguistic groups of Kwa are geographically disseminated in southern and middle Benin and represented by Adja, Houeda, Sahoue ethnic groups and relatives, Mina, Anii, Windji-windji ethnic groups and relatives, Fon, Mahi, Goun, Tofin, Xwla ethnic groups and relatives. The socio-linguistic groups of Yoruboїd are mostly found in middle and southeastern Benin with Yoruba, Idashaa, Nagot ethnic groups and relatives. The socio-linguistic groups of Gur are located in northern Benin and include Bariba, Ditamari, Berba, waama ethnic groups and relatives, Gurma, Natimba ethnic groups and relatives, Lokpa, Coto-coli, Kabye ethnic groups and relatives, Yom, Yoa Taneka ethnic groups and relatives [60].

### Sampling and data collection

These data are part of a large database collected by students of master degree in the framework of a research project on HGs in Benin. The sampling strategy developed (see also [22, 37]) included probabilistic and non-probabilistic approaches. Firstly, a rapid rural appraisal approach was carried out with agricultural extension services and was used to identify 3 districts of interest in each bio-geographical zone: Tanguiéta, Boucoumbe and Toucountouna in the semi-arid zone, Bassila, Dassa and Bantè in the sub-humid zone and Aplahoué, Agbangnizoun and Zogbodomey in the humid zone. An exploratory survey was conducted on 60 randomly selected informants in each district. We defined informant as the household member with extended rights on the HG including management decision, composition, right to sale products from gardens but not including necessarily land property right. The exploratory survey was intended to determine the proportion of HG owner per bio-geographical zone and consequently the sample size (n) in each biogeographical zone using the normal approximation of the binomial distribution (Dagnelie 1998):

$$ \mathrm{n}=\frac{{\mathrm{U}}_{1\hbox{-} \upalpha /2}^2\times \mathrm{p}\left(1\hbox{-} \mathrm{p}\right)}{{\mathrm{d}}^2} $$

*U*_1 − *α*/2_^2^ is the value of the Normal random variable at probability value of 1 − α/2. For a probability value of 0.975 (or α = 0.05), *U*_1 − *α*/2_^2^ ≈ 1.96; *d* is the margin error of the estimation of any parameter to be computed from the survey and a value of 8 % [37] was considered.

Values of *n* were rounded to 75 in the Semi-arid zone and to 80 in the humid and sub-humid zones. An equivalent of *n* for non-HGs owners was also selected in each zone. Informants interviewed during the depth survey and gardens inventories were taken from the list of informants (owner of HG or not) previously selected in each bio-geographical zone (exploratory survey). Snowballing approach was used to select additional informants when the list was exhausted. As a result, 470 informants were sampled for individual interviews including 235 HGs owners and 235 non- owners. Interviews generally lasted 60 to 90 min when the researcher could communicate directly with the informants, and more time (~120 minutes) whenever assistance of a translator or other relevant informant was required. Interviews were recorded primarily using a questionnaire. Additionally, a digital recorder was also used to record all exchanges with informants when necessary.

Field work took place between June and November 2011 coinciding with cultivation periods and growing phase for vegetation. During these periods, wild and crop trees, shrubs and herbs are more visible and easily identifiable.

Following the age categorization used by Idohou et al. and Assogbadjo et al. [37, 64], 24.07 % of the informants were young (age < 30), 59.30 % were adult (30 < age < 60) and the remaining 16.63 % were old people (age > 60). The three major socio-linguistic groups in Benin were represented as follows: Kwa (37.20 %), Gur (40.92 %) and Yoruboїd (21.88 %). Approximately one third of informants were female (36 %). Regarding education level, 40 % were uneducated, 32 % attended primary school or Alphabetized classes and 28 % attended secondary school or more. Agriculture including crop production and small scale livestock was the main activity of 60 % of the informants; the others being mainly engaged in services (Teaching, craft, motorbike taxi, etc.).

While the demographic data i.e. Age, Gender, Education level and Main economic activity were collected on all the respondents to analyze their effect on HG ownership, only the 235 HGs owners were retained for diversity and structure analyses in HGs. Their HGs were visited and an exhaustive floristic inventory was carried out. Inventoried species were identified and named following the Botanical nomenclature of Lebrun and Stork [65]. Vouchers of species that could not be identified in field were sent to the national herbarium of Benin where they have been identified by botanists using the national collection database and taxonomic keys. They have been helped by a short description attached to each voucher (local name, site of collection etc.). All vouchers were identified to species level.

The total number of individuals as well as the area covered by each species in a HG were recorded. The covered area of each species was recorded as the abundance/dominance coefficient following Braun-Blanquet [66]. Areas of HGs were measured and their ages were estimated with the assistance of tenders. For each HG, it was also noted if the HG has been inherited or set up by the owner.

### Data analysis

#### Assessing the socio-economic factors influencing HG ownership

A generalised linear model (GLM) with binomial error distribution, was used to assess the socio-economic factors influencing HG ownership (*Yes* coded 1 or *No* coded 0). The used explanatory variables included gender (*Male* versus *Female*), education level (*Uneducated*, *Primary school*, *Secondary school and more*) and main economic activity (*Agriculture and non-agriculture*) as factors and age as covariate. The same statistical method was used to explore the effect of gender and age of HG owners on origin of HG (Inherited versus Non-inherited). Specifically for *non-inherited* HG i.e. own established HG, we performed a GLM with negative binomial error to assess the effect of age and gender on their occurrence. This enables inferring the temporal pattern of establishment of HG across gender. A negative binomial error was preferred over a poisson or quasi-poisson error because it showed the lowest deviance [67].

#### Assessing socio-economic and HGs characteristics influencing plant diversity in HGs

Species richness of each HG was used as an estimate of plant diversity in HG. The socio-economic characteristics used were the same as above (i.e. age, gender, education level and main economic activity) while HGs characteristics included HG size and age, which were considered as covariates. A GLM with the negative binomial error distribution that showed the lowest deviance [66] was used to investigate which of the above characteristics significantly affected plant diversity in HGs.

For all GLMs, only significant terms or terms included in a significant interaction were retained in the final model. To this end, all possible interactions were first included in the initial models. These models were then simplified in a stepwise-backward procedure coupled with chi-square (χ2) tests based on likelihood ratio to obtain minimal adequate models.

#### Assessing structure of home gardens and their relationship with socio-economic conditions of HG owners

To assess structure of HGs and their relationship with socio-economic conditions of HGs owners a four step analysis was conducted. The encountered plant species were first classified into five plant morphological groups (life form) including Herbs, Shrubs, Lianas, Palms and Trees. In a first step, a hierarchical clustering (using Ward distance) of HGs was performed based on the following HG structural characteristics: HG size, species richness (SR), number of CWRs (CWR), raw spectrum (RS) and weighted spectrum (WS) of each of the five plant groups, i.e. RS-Herbs, RS-Shrubs, RS-Liana, RS-Palms, RS-Trees and WS-Herbs, WS-Shrubs, WS-Liana, WS-Palms, WS-Trees respectively. Both RS and WS were expressed in %. RS measures abundance while WS measures dominance. This analysis enabled to define ten clusters of HGs. A matrix presenting the mean of the above structural parameters per cluster of HGs were thus defined. In a second step, a principal component analysis was used on this matrix to describe on the one hand the relationships between the structural parameters of HGs and on the other hand the structure of each cluster. This description allows categorizing again the HG clusters into three categories. In a third step, the relative frequencies of the categories of HGs were assessed across bio-geographical zones to detect whether prevalence of the categories of HGs varied across bio-geographical zones. Finally, fifteen groups of HG owners were defined by the combination of the levels of the socio-economic factors: age category (Young, Adult, Old), gender (Male, Female), education level (Uneducated, Primary school level, Secondary school level) and main economic activity (Agriculture, Non-agriculture). Fifteen groups were defined instead of 36 because some categories lack HG owners. A contingency table was built by crossing these categories of socio-economic characteristics and the three categories of HGs. A simple correspondence analysis was then used on this contingency table to examine relationships between the categories of HGs and socio-economic characteristics of HG owners (age, gender, education level and main economic activity).

Packages *MASS* [68] and *FactoMineR* [69] of the statistical software R version 2.15.3 (R Development Core Team 2013) were used for the negative binomial GLM and the multivariate analyses respectively. Statistical significance was set to 5 %.

## Results

### Socio-economic factors influencing HG ownership

Regardless of their status (HG owner or not), informants interviewed were mostly male (64 %) but there were more females among informants with home gardens than informants without home gardens (respectively 44 % and 29 %). The proportion of adult and old people (age >30) was higher among HG owners as compared to non-owners (88 % vs. 64 %). Farming was the predominant activity among informants regardless of HG ownership (61 % and 56 %, respectively for owners and non-owners). Respectively 37 % and 44 % of HG owners and non- owners were uneducated (never attended the school). Among the four explanatory variables abovementioned (gender, age, education level and main economic activity), only three i.e. gender, age and education level were retained in the model after stepwise selection. Furthermore, only the “age of HG owner” was found to significantly determine ownership of HG (*p-value* < 0.001, Table 2). Regardless of gender and education level, age is positively correlated with HG ownership (0.094; Table 2, Fig. 2a). Significant interactions included age ~ gender (*p-value* <0.026), age ~ education level (*p-value* <0.014) and age ~ gender ~ education level (*p-value* <0.002) (Fig. 2a, Table 2), indicating that the effect of age was respectively gender-dependent, education level-dependent and both gender and education level dependent. Irrespective of education level, it was more likely to find a female owner than a male owner with increasing age (Table 2, Fig. 2b). But the reverse case was true beyond 70 years old (Table 2, Fig. 2b). Similarly, with increasing age and irrespective of the gender, most owner are educated at primary school level (Table 2, Fig. 2c). Finally, with increasing age, the uneducated informants and those with primary school education contained more males than females HG owners, while among the informants of secondary school level or more, there were more females than males HG owners (Table 2, Fig. 2d).

**Table 1:** Characteristics of the three bio-geographical zones

	Bio-geographical zones		
Parameters	Semi-arid	Sub-humid	Humid zone
Location	9°45’-12°25’ N	7°30’-9°45’ N	6°25’-7°30’ N
Rainfall regime	Unimodal	Unimodal	Bimodal
Rainfall (mm)	<1000	900-1110	1200
Temperature (°C)	24-31	25-29	25-29
Relative humidity (%)	18-99	31-98	69-97
Climate type	Dry tropical	Humid tropical	Humid tropical
Density of population^††^	33-49	51-162	191-8593
Days of growing season	90-100	180-270	270-365

Adapted from Jahnke and Jahnke [57] Natta et al. 2003; Hijmans et al. 2004; Sinsin et al. [58]. INSAE [59] ^††^Inhabitant.km^−2^

**Table 2:** Socio-economic factors influencing HG ownership: summary of the results of the generalised linear model using the negative binomial error distribution (R^2^ = 0.18)

Factors	Estimate	Std. error	z-value	Pr(>|z|)
Male	0.523	0.757	0.691	0.489
Age of HG owner	0.094	0.028	3.418	**0.001**
Schol+	−1.106	0.893	−1.238	0.216
Unedu	0.247	0.814	0.303	0.762
Male:Age of HG owner	−0.053	0.024	−2.219	**0.026**
Age of HG owner:Schol+	0.044	0.033	1.344	0.179
Age of HG owner:Unedu	−0.061	0.025	−2.464	**0.014**
Male:Age of HG owner:Schol+	−0.033	0.021	−1.590	0.112
Male:Age of HG owner:Unedu	0.046	0.015	3.059	**0.002**

*Schol+* secondary school and more, *Unedu* uneducated

**Fig. 1:**
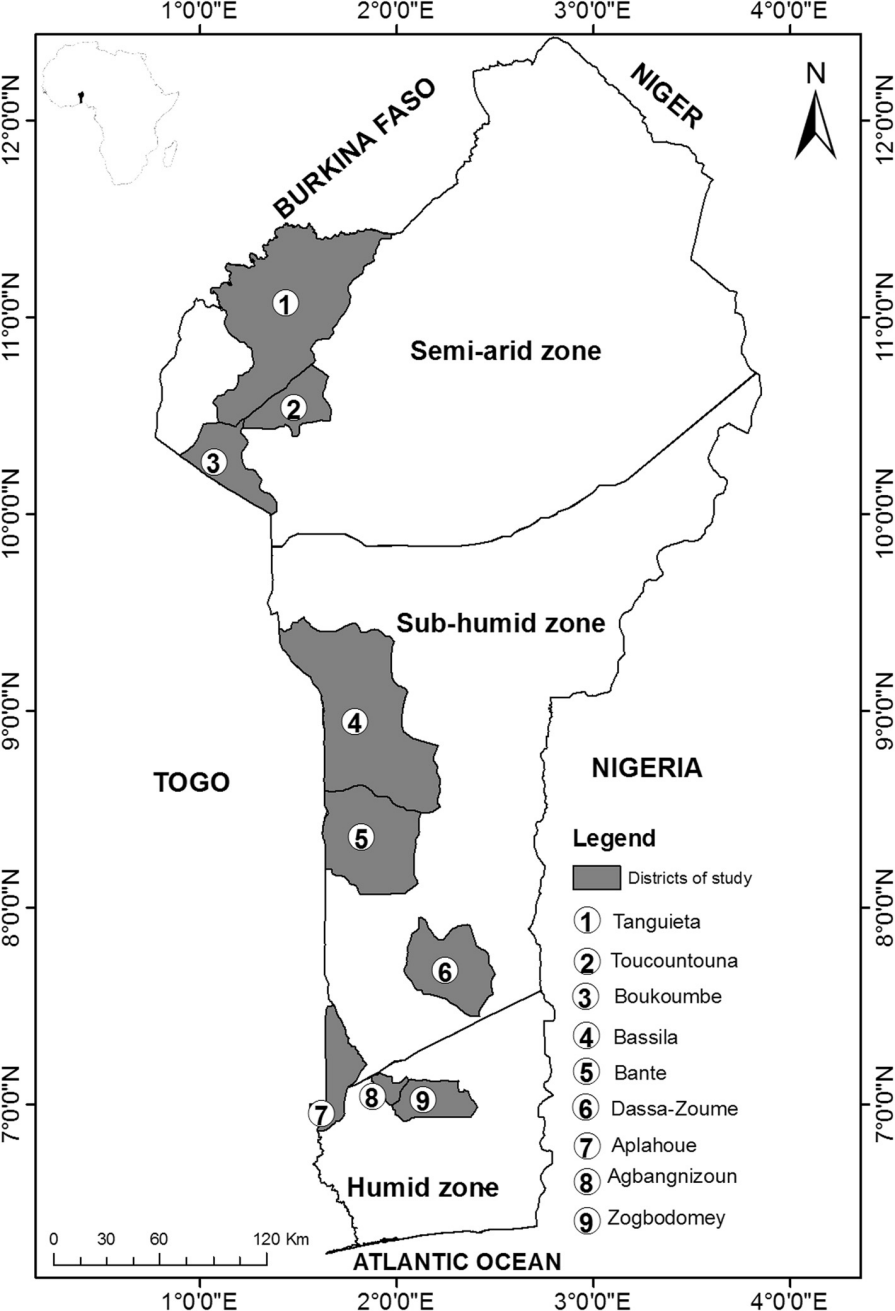
Location of study sites

**Fig. 2:**
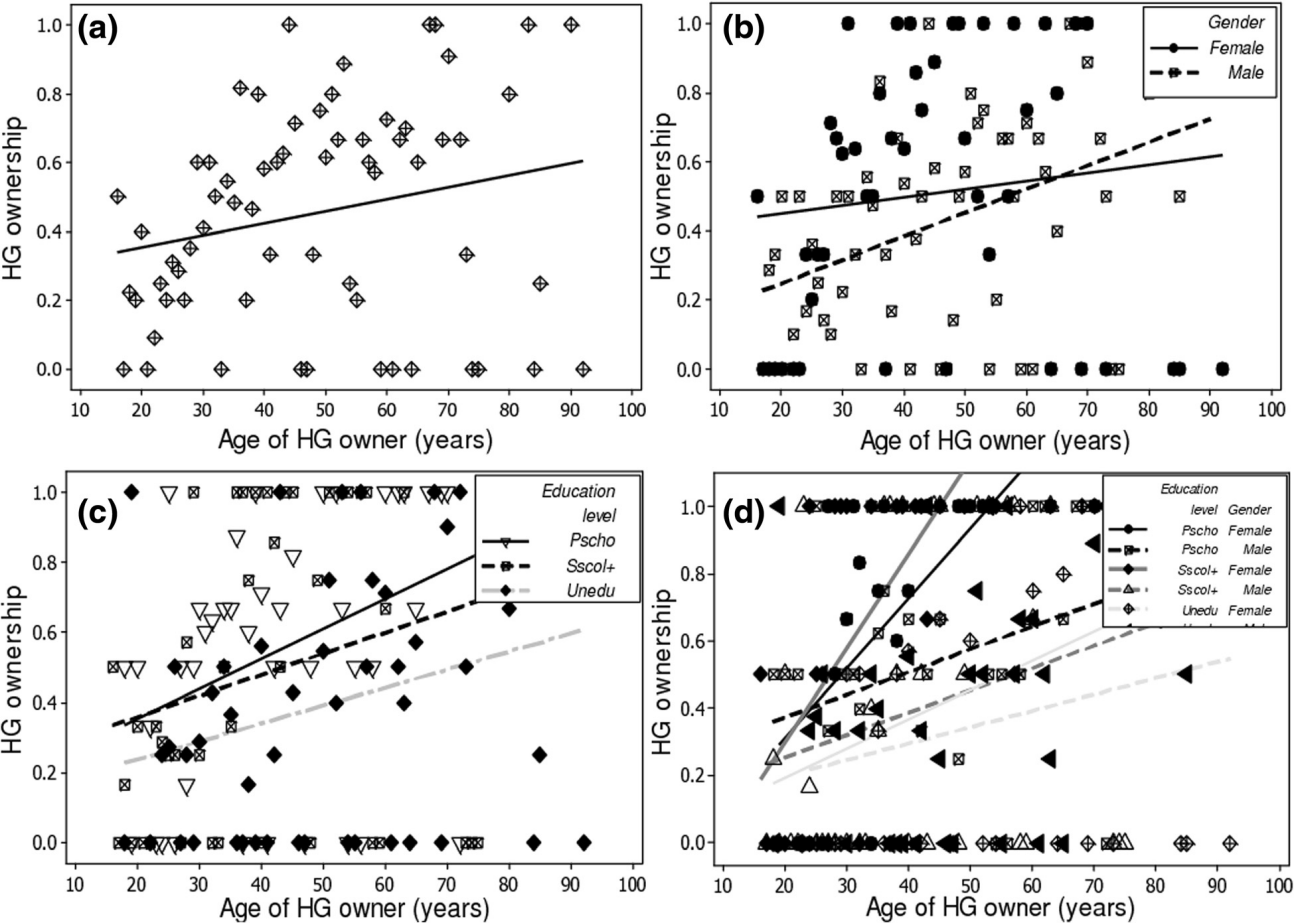
Effect of age, gender, education level and their interactions on HG ownership

**Fig. 3:**
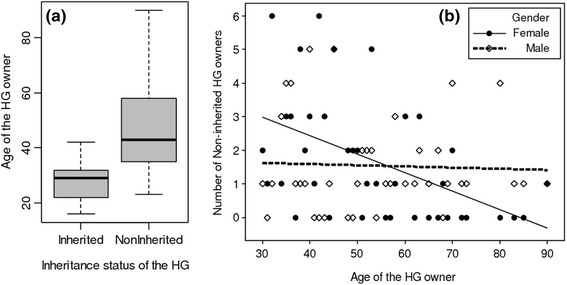
Effects of age and gender of owners and the inheritance status of HG

When examining the origin of the HG, only age was found to significantly affect inheritance status of HG (GLM with negative binomial error distribution, Df = 1, Deviance = 34.68, Prob. = 0.000, R^2^ = 0.41). Inherited HGs were owned by young informants while non-inherited HGs i.e. self-established HG were owned by adults and older people (Fig. 3a). Examining occurrence of non-inherited HGs against age and gender of their owners, we found that these two factors non-additively significantly affect number of non-inherited HGs in addition to the significance of their main respective effects which together explained 25 % of the observed variation (Table 3). While number of non-inherited HGs decreased with increasing age of HG owner for females, it was stable for males (Fig. 3b).

**Table 3:** Effect of age and gender on number of Non-inherited HGs: summary of the results of the generalised linear model using the negative binomial error distribution (R^2^ = 0.25)

Factors	Estimate	Std. error	z value	Pr(>|z|)
(Intercept)	2.562	0.550	4.654	0.000
Age	−0.041	0.011	−3.697	0.000
Gender Male	−2.009	0.764	−2.630	0.008
Age: Gender Male	0.039	0.015	2.670	0.007

### Socio-economic conditions and HG features influencing plant diversity in HGs

285 plant species were recorded in the 235 inventoried home gardens spanning the three bio-geographical zones. The average number of species per home gardens was 10.1 species with a coefficient of variation of 58.15 % indicating some discrepancies among HGs. The richest HG hosted 52 plant species, whereas the poorest HG held one plant species. The average value of plant diversity was 9.97 ± 1.03 SE species for young, 10.23 ± 0.50 SE species for adult and 10.17 ± 0.80 SE species for old HGs owners. Similarly, the average value of plant diversity was 9.81 ± 0.46 SE species for women HG owners versus 10.48 ± 0.59 SE species for men.

**Fig. 4:**
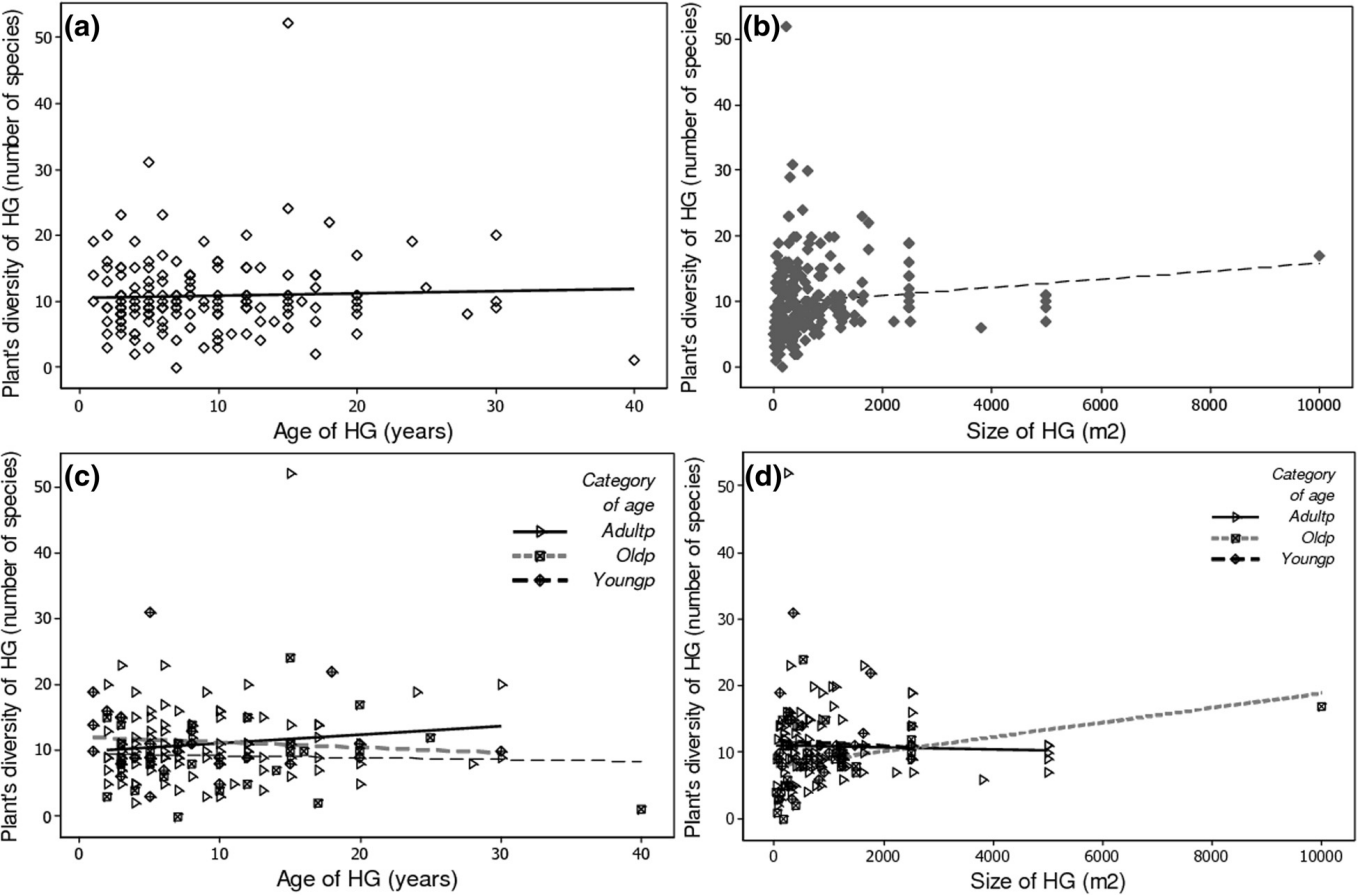
Effect of the age and size of HGs and their interaction with the age of the HG owner on plant species richness of HGs YoungP = Young people; Adultp = Adult people; Oldp = Old people

Neither the age of HG owner nor the size of the HG significantly influenced plant diversity richness in HGs. However, age of HG overall significantly (*p-value* < 0.033) determined plant species richness in HGs. The positive value of the estimate for this factor (0.035, Table 4) indicate that the older the HG, the more diversified it is (Fig. 4a). Plant diversity fairly increased with increasing size of HG (Fig. 4b) without being significant (*p-value* < 0.053). Significant interactions were also found for *age of HG owner ~ size of HGs (p-value* < 0.023) and *age of HG owner ~ age of HGs (p-value* < 0.036) (Table 4), indicating that the effect of the size of HG and the effect of the age of HGs on plant diversity in HGs were dependent on the age of the owner. With increasing age of HG, the cultivated plant diversity decreased for young, increased for adult and was stable for old owners (Fig. 4c). With increasing size of HGs, plant diversity decreased for young and adult person but increased for old persons (Fig. 4d).

**Table 4:** Socio-economic factors and HGs characteristics influencing cultivated plant diversity in HG: summary of the results of the generalised linear model using the Poisson error distribution (R^2^ = 0.11)

Factors	Estimate	Std. Error	z value	Pr(>|z|)
Age of HG owner	−0.0021	0.0042	−0.510	0.610
Size of HG	−0.0002	0.0001	−1.931	0.053
Age of HG	0.0349	0.0164	2.133	**0.033**
Age of HG owner: Size of HG	0.0000	0.0000	2.269	**0.023**
Age of HG owner: Age of HG	−0.0007	0.0003	−2.098	**0.036**

*HG* home garden

### Structure of HGs and its relationships with socio-economic characteristics of HG owners

#### Structure of HGs

The hierarchical cluster analysis applied on the structural floristic characteristics of the 235 HGs, distinguished 10 clusters of HGs. This was defined by their size, plant diversity (species richness), richness of crop wild relatives (CWR), and prevailing plant group. Cluster 1 grouped essentially herb based gardens (respectively 89.03 % and 94.86 % of raw and weight spectra) with an average size of 0.09 ha ± 0.01 SE. Average species richness was 9.22 ± 0.75 SE, including 0.45 ± 0.10 SE CWRs. Cluster 2 grouped small size HGs (0.02 ha ± 0.01 SE), hosting on average 7.38 species ± 0.70 SE including 0.02 ± 0.10 SE CWRs. This cluster is composed mostly of herbs (respectively 57 % and 87.68 % of raw and weight spectra), but also including shrubs (respectively 28.79 % and 8.76 % of raw and weight spectra) and trees (respectively 13.74 % and 3.48 % of raw and weight spectra). Home gardens under Cluster 3 showed even predominance of herbs (respectively 40.54 % and 51.94 % of raw and weight spectra) and shrubs (respectively 39.39 % and 40.1 % of raw and weight spectra) but also trees (respectively 18.70 % and 7.35 % of raw and weight spectra). The average size was 0.04 ha ± 0.01 SE for an average species richness of 14.34 ± 0.09 SE including 0.55 ± 0.09 SE of CWRs. Cluster 4 gathered gardens mainly composed of herbs (55.66 % and 69.94 % respectively of raw and weight spectra) but also of shrubs (respectively 18.71 % and 21.88 % of raw and weight spectra), palms (respectively 17.89 % and 5.39 % of raw and weight spectra) and few trees (7.74 % and 2.80 % respectively of raw and weight spectra). The average size was 0.05 ha ± 0.00 SE with a species richness of 7.19 ± 0.62 SE including 0.56 ± 0.04 SE CWRs. Cluster 5 also included herb-based gardens (respectively 70.68 % and 78.83 % of raw and weight spectra) yet with notable prevalence of trees (respectively 21.75 % and 14.50 % of raw and weight spectra) and few shrubs (6.27 % and 6.07 % respectively of raw and weight spectra). The average size was 0.05 ha ± 0.01 SE with a species richness of 12.10 ± 0.59 SE including 1.14 ± 0.11 SE CWRs. Cluster 6 grouped gardens mostly composed of herbs (57.44 % and 72.03 %), shrubs (respectively 16.64 % and 13.40 % of raw and weight spectra), trees (respectively 14.29 % and 9.79 % of raw and weight spectra) and Liana (respectively 11.31 % and 4.75 % of raw and weight spectra). These HGs in average covered 0.11 ha ± 0.02 SE with a species richness of 10.68 ± 1.08 SE including 0.50 ± 0.06 SE CWRs. In contrary to previous clusters, shrubs (respectively 43.85 % and 72.08 % of raw and weight spectra) were predominant in HG of cluster 7 which also hosted trees (respectively 27.29 % and 4.13 % of raw and weight spectra) and herbs (respectively 26.65 % and 23.25 % of raw and weight spectra). These gardens were in average of small size (0.02 ha ± 0.00 SE) with a species richness of 6.12 ± 0.86 SE including 0.12 ± 0.08 SE CWRs. Cluster 8 grouped gardens composed mostly and almost equally of shrubs (respectively 39.23 % and 33.82 % of raw and weight spectra) and herbs (respectively 37.28 % and 33.82 % of raw and weight spectra) but also including a few trees (respectively 17.03 % and 13.46 % of raw and weight spectra) and few Liana (respectively 3.62 % and 1.63 % of raw and weight spectra) and palms (respectively 2.85 % and 1.63 % of raw and weight spectra). These gardens covered in average 0.04 ha ± 0.01 SE with a species richness of 31.5 ± 04.31 SE including 0.83 ± 0. 17 SE CWRs. The two last clusters were particularly different from the previous. Cluster 9 was indeed composed mostly of palms (respectively 54.4 % and 50.88 % of raw and weight spectra) and Liana (respectively 18.02 % and 39.54 % of raw and weight spectra) but also included shrubs (respectively 19.315 and 8.96 % of raw and weight spectra), a few trees (6.77 % and 0.01 % respectively of raw and weight spectra) and few herbs (respectively 1.47 % and 0.62 % of raw and weight spectra). The average size was 0.18 ha ± 0.05 SE with a species richness of 11.38 ± 1.34 SE including 0.37 ± 0.18 SE CWRs. Finally, cluster 10 grouped palm-dominated HGs (respectively 64.43 % and 90.68 % of raw and weight spectra) but also hosting shrubs (respectively 24.04 % and 6.38 % of raw and weight spectra) and few herbs (respectively 4.45 % and 1.61 % of raw and weight spectra), trees (respectively 4.19 % and 0.00 % of raw and weight spectra) and Liana (respectively 2.90 % and 1.13 % of raw and weight spectra). The average size was 0.4 ha ± 0.08 SE with a species richness of about 10.10 ± 0.85 SE including 0.2 ± 0.13 SE CWRs.

Overall, the five first clusters encompassed approximately 73.7 % of the sampled HGs while the remaining clusters encompassed 26.3 % of the sampled HGs. Clusters 2, 3, 4, 5, 7 and 8 contain small size HGs (0.02 to 0.05 ha), while clusters 9 and 10 contain the largest HGs(0.18 to 0.4 ha). The remaining clusters encompassed HGs with intermediate size ranging in average from 0.09 to 0.11 ha. Most clusters included HGs with an average plant richness ranging from approximately 7 to 12 species. The highest values of plant richness were found in HGs of cluster 8 with a maximum of 52 species. The HGs of all the clusters portrayed relatively low (if any) richness of CWR. Higher values of CWR (2 to 4) and were observed in HGs of clusters 1 and 5. Based on prevailing plant groups, clusters 1, 2, 4, 5 and 6 were those of HGs with high prevalence of herbaceous plants while clusters 7 and 8 were gardens with high prevalence of shrubs and trees. Clusters 9 and 10 contain gardens with high prevalence of palms and Liana.

**Fig. 5:**
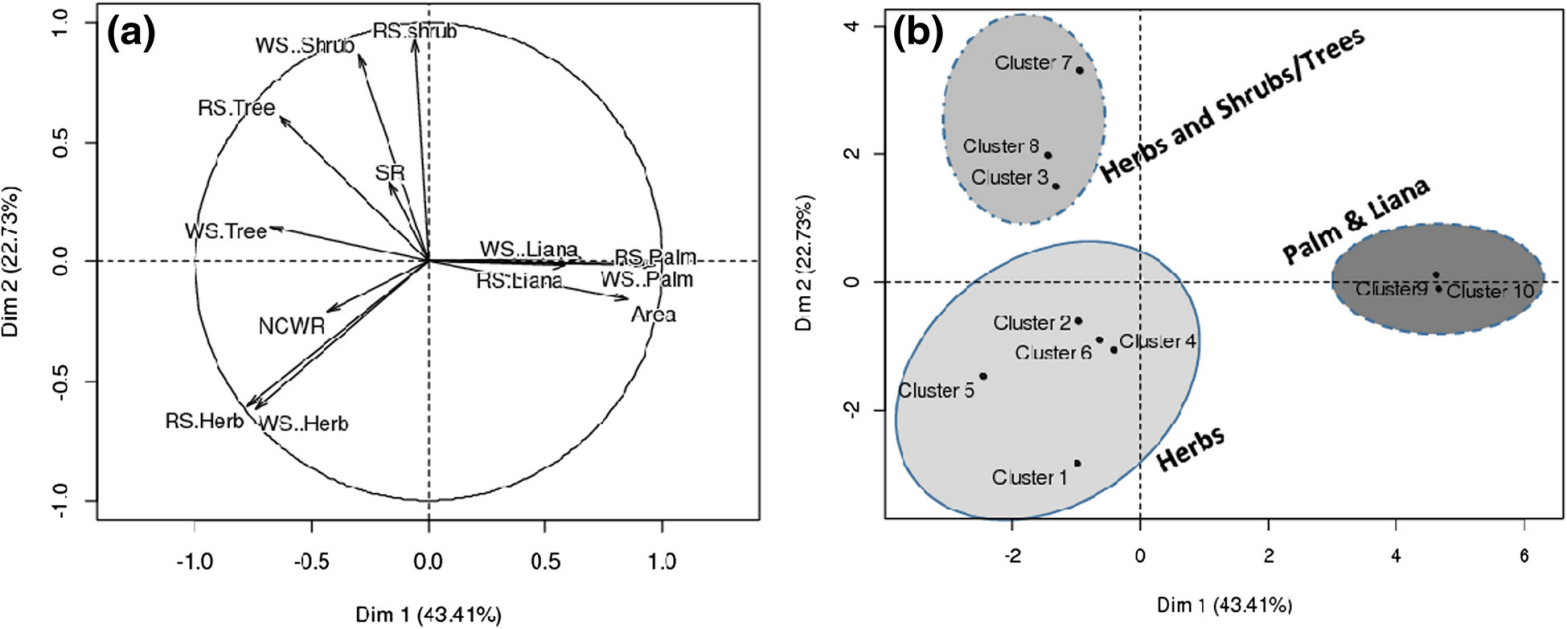
Projection of structural features of clusters of HGs (**a**) and projection of clusters of HGs (**b**) in the axes system1 and 2. RS = Raw Spectrum; WS = Weighted Spectrum; Lia = Liana

Results of the principal component analysis (PCA) on the structural parameters of HGs by the previous clusters (Fig. 5) saved 66.14 % of the total variance on the two first components. The first component is positively correlated with size of HGs, prevalence of palms and lianas whereas it is negatively correlated with richness of CWR, herbs and tree prevalence (Fig. 5a). The second component is positively correlated with tree and shrub prevalence and negatively correlated with size, richness of CWR and Herb prevalence (Fig. 5a). Thus, Palm and Liana prevailing HGs were often larger with low richness of CWR and to a lesser extent with low overall plant richness. In contrast, HGs with a high number of herbs and/or shrubs were often of small size. However, large herbs-dominated HGs were often floristically more diversified and showed higher richness of CWR.

Plotting the ten clusters onto the two axes, the three previous main categories of HGs were easily distinguishable (Fig. 5b).

- **Herb-based HGs,** represented by clusters 1, 2, 4, 5 and 6 (Fig. 5b). Within this category, HGs areas were highly variable ranging from 0.01 ha to 0.38 ha. The plant richness was among the highest of the sample for larger herb-based gardens but low for smaller herb-based gardens. Smallest HGs of this category sheltered no CWR while large gardens (i.e. cluster 5) hosted the highest CWR richness;
- **Herbs and shrubs/trees-based HGs,** represented by clusters 3, 7 and 8 (Fig. 5b). Within this category, HGs were of smaller sizes with also plant diversity amongst the smallest of the sample with very few if none CWR recorded. However, some HGs of this category (i.e. Cluster 3) were exceptionally richer. HGs of this category were also characterised by notable presence of trees;
- **Palm and liana-based HGs,** represented by clusters 9 and 10 (Fig. 5b). These HGs were the largest, yet with low overall plant and CWR richness.

#### Structural characteristics of HGs across bio-geographical zones

**Fig. 6:**
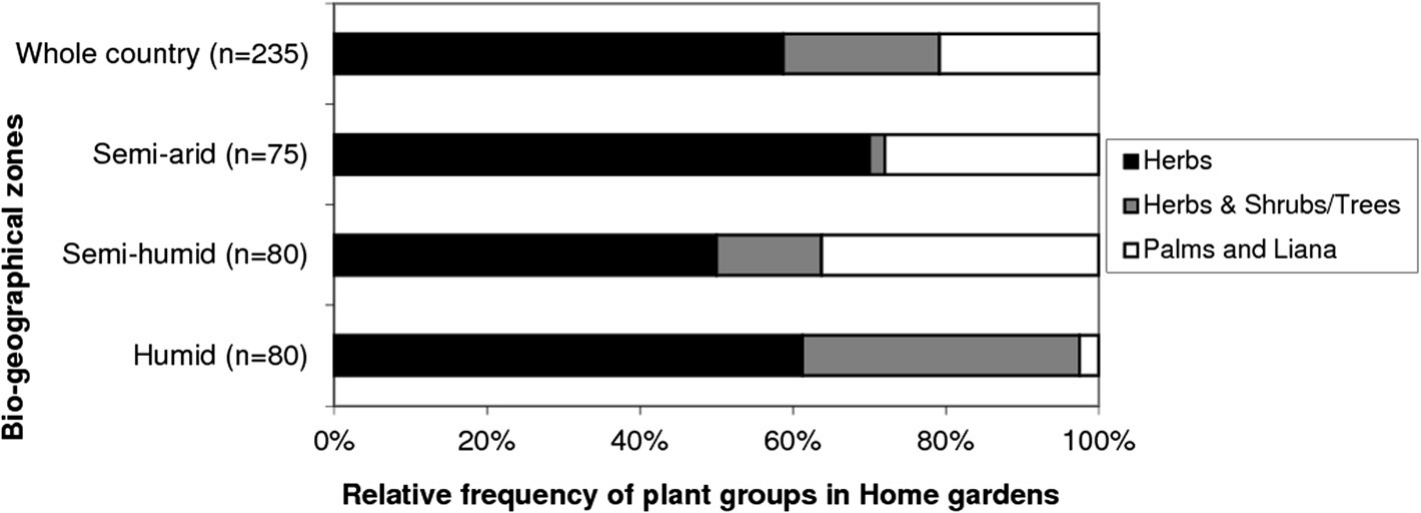
Relative frequency of home gardens with high prevalence of specific plant group across bio-geographical zones

Clusters of HGs were diversely distributed across the three studied bio-geographical zones. Clusters 3, 4, 5, 6, 7 and 8 were only encountered in humid and sub-humid zones while clusters 1 and 2 were only observed in semi-arid zone. Clusters 9 and 10 were observed in sub-humid and semi-arid zones. Regarding the prevailing plant group, herb-based HGs were the most prevalent in general, although this varied depending on the bio-geographical zone (Fig. 6). Herbs and shrubs based, shrubs and trees based, and herb and trees based gardens were most often encountered in humid and sub-humid zones and rare in the semi-arid zone. Palm and liana-based gardens were often encountered in sub-humid and semi-arid zones.

Analysis of the vertical arrangement of the vegetation within gardens revealed that most gardens (96.75 %) contained an herbaceous stratum while about 50 % of them had an intermediate shrub layer and 32 % an upper stratum (Tree or palm). Most HGs in the humid zone lacked an upper tree layer (85.21 % of gardens). HGs with an intermediate layer were mostly encountered in humid (44.5 % of gardens) and in sub-humid HGs (70.5 % of gardens). Gardens with upper stratum were mostly observed in sub-humid (45.75 % of gardens) and Semi-arid zones (38.5 % of gardens). Some rare cases (3.25 %) of HGs without herbaceous stratum were encountered in the sub-humid zone.

#### HGs structure as related to socio-economic characteristics of HG owners

**Fig. 7:**
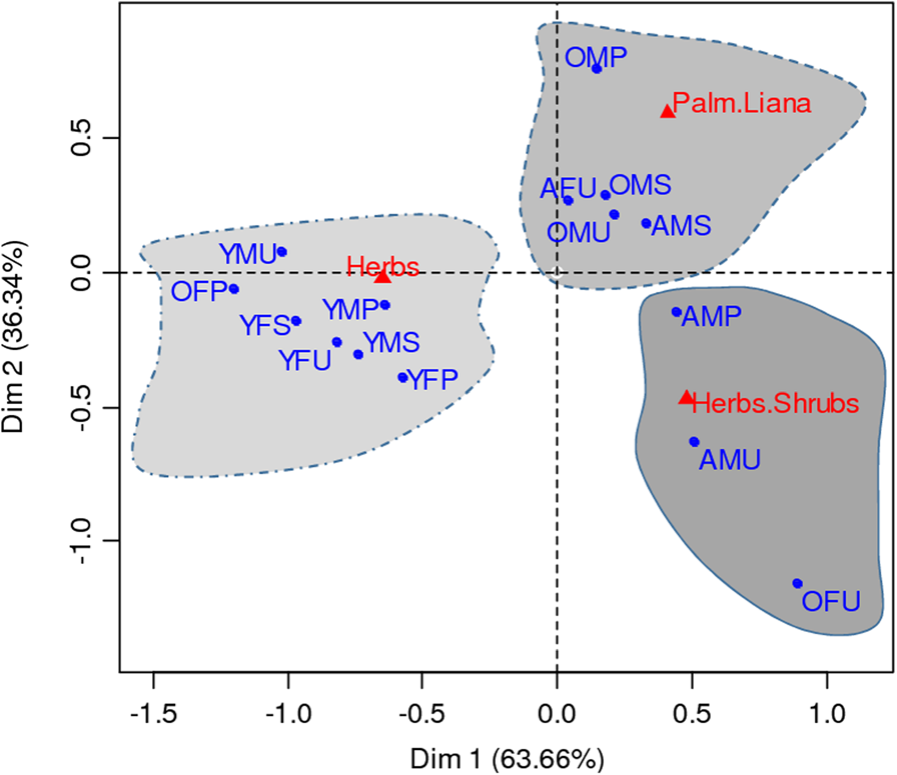
Projection of HGs categories and HGs owner socio-economic characteristics in the systems axis 1 and 2 HGs categories: *HHG = Herb based Home garden, HSHG = Herb and Shrub based Home Garden, SHG = Shrub based Home Garden, TPHG = Tree and Palm based Home garden*−−Socio-economic groups of HGs owners: *YMU = Young Male Uneducated, YMP = Young Male with Primary school level, YMS = Young Male with Secondary school level, YFU = Young Female Uneducated, YFP = Young Female with Primary school level, YFS = Young Female with Secondary school level, AMU = Adult Male Uneducated, AMP = Adult Male with Primary school level, AMS = Adult Male with Secondary school level, AFU = Adult Female Uneducated, AFP = Adult Female with Primary school level, AFS = Adult Female with Secondary school level, OMU = Old Male Uneducated, OMP = Old Male with Primary school level, OMS = Old Male with Secondary school level, OFU = Old Female Uneducated, OFP = Old Female with Primary school level, OFS = Old Female with Secondary school*

The correspondence analysis assessing how HGs structure is related to socio-economic characteristics of HG owners (Fig. 7) indicated (with 100 % of variance saved) the following:

- **Herb-based HGs** were owned mainly by young men as well as young, adult and old women, regardless of the education level (Fig. 7). Small HGs were owned by young men and women while larger HGs were owned by adult and old women.
- **Herbs and Shrubs based HGs** belonged mainly to men regardless of age and education level (Fig. 7).
- **Palms based HGs** were owned mainly by old men regardless of education level and also by adult men with primary and secondary level school and adult uneducated women (Fig. 7). Gardens owned by old men were larger, more diversified and with higher CWR record.

## Discussion

This study analyses how socio-economic conditions of people and HGs characteristics determine ownership, plant diversity as well as structure of HG.

The fact that probability of HG ownership increases with age suggests that adult and elderly people are more involved in gardening activities than young people. This finding supports previous observations [14] in Nicaragua, [70] Texas (USA) and [71] central Italia. Such finding, particularly for old persons could suggest that gardening activities do not require intensive physical efforts and thus are adapted to declining capacity of old people to reach distant farms or/and need to have useful plants species at hand for daily use. In the particular context of the study area, women and older men are known to keep cultivation fields close to their village including HGs, while the younger men rather use distant fields [72].. While there was no discrepancy in HG ownership between adult men and women, at an earlier age (<30), it was more likely to find young women owner of gardens than young men and inversely at a later age (>60). Early involvement of women in home gardening is consistent with the trend previously observed in Latin American HGs [14, 18, 52]. Such observation could be explained by the local and cultural context of the study area, which predisposes women at earlier age to home gardening. Indeed, there is a discrepancy between young men and women in access to school [73] with less enrolment for young women. Thus the latter are more involved in household related works than younger men. The early involvement of young women in gardening activities is probably not a deliberate choice but rather a strategy to gain a certain social status, enhance their livelihoods and purchase power [18], particularly in a patriarchal context of African societies where farmland property right is largely male-dominated [74]. Our data also support the hypothesis that the education level does not affect HG ownership. This observation is not necessarily associated with the low school enrolment rate of developing countries. For instance in a case study from a developed country (Texas, USA) [70] HG owners were also irregularly distributed among education levels with no obvious trend. However, we remarked that more HG owners were less educated, probably because people with higher education level have access to other job opportunities and thus may have less time to allocate to home gardening.

In the study area we detected two ways to own a HG: non-inheritance i.e. own initiation and inheritance. Inheritance of HG was consistently more seen in young informants than for adults and old informants.

Because only 18 % of the observed variation in HG ownership was explained by age, gender and education level, in the future additional characteristics (i.e. status in household, farmland asset, distance from farm, number of farms, etc.) as well as local context (i.e. importance of animal rearing, Land tenure, market opportunities, etc.) should be combined to develop reliable predictive models of HG ownership.

Plant diversity in HG is assumed to be determined by complex socio-economic and ecological factors [49, 50] as well as intrinsic features of HG [75, 76]. While our data partially support this hypothesis, our analyses suggest that this should be placed into a specific context. For instance in this study neither the age of gardener, gender, education level nor size of HG were found to significantly influence plant diversity (species richness) in HGs. These observations are inconsistent with previous studies in tropical HGs. Indeed age and gender of the garden caretaker were previously reported to be significant variables explaining differences in agro-biodiversity among households in the Peruvian amazon [77] where an increase of 10 years in age corresponded to a predicted increase of 1.40 species in the garden. Also, garden size significantly influences species richness in different regions worldwide (e.g. Niger [76]; Northeastern Brazil [75]; Indonesia [78]). While these trends were not observed in Benin, our data rather revealed that the age of the HG significantly determined its plant diversity (*p-value* < 0.033). The older the HG, the more diversified the species (richness) (Table 3, Fig. 5), which is congruent with observations in Amazonian villages [49] but contradicts findings in Mexico [52] and in Indonesia [53], where plant diversity in young HGs was higher than in older ones. Beyond differences in socio-economic and ecological contexts among these regions, the discrepancies observed in this study would suggest that (i) the age of HG is not a comprehensive and reliable predictor of plant diversity in HGs and (ii) it should be considered in its context, for instance by accounting for the age of the gardener. Indeed, our findings support that plant diversity in HGs is determined by interaction of age of HG with age of gardener. While plant diversity within HGs decreased with the age of HG for young owners, it increased with the age of HG for adult and was stable with age of HG for elderly people. These results could be explained by the following two reasons. First, young people tend to produce plants with attractive market value [38]. As such, diversity of plant within their HGs would continuously be adapted to local market demand. Second, young people who generally inherited their HGs may have little knowledge about all plants maintained within these HGs. Consequently they could selectively use and manage plants of interest and then cause depletion of unknown plants. In contrary, adult people might be less market-oriented and therefore cultivate plants regardless of their cash value.

Plant diversity in HG was also influenced by the interaction of the size of HGs and age of the gardener. Indeed, plant diversity decreased with increasing size of HG for young and adult persons and increased with increasing size of HG for the elderly. Such observations indicate a trend of simplification of home gardens diversity with increasing HG size across generations and could suggest a critical decline of the plant diversity maintained in HG across generations. Indeed, the age of the owner of the HG is very important because plant acquisition is known to be a lifelong undertaking [77].

Only 11 % of the total variation of plant diversity among HG was explained by the here-considered variables (age, gender, education level, age and size of HG) suggesting that there is a need to integrate supplementary factors such as land assets, social capital, culture and labor into the model [77]. Additionally, there is a need to better understand the function of a HG in a specific context. While their contribution to agro-biodiversity conservation is obvious [20, 22], in many cases, HGs are primarily devoted to food and non-food productions [11]. With agricultural dynamics including production orientation, changes on HGs structure have been reported [79, 41]. In the study area some gardens were converted into “small farms” for conventional herb of vegetables production (Gbedomon R.C., field observations). Personal observations, which should be tested in the future, suggest that models explaining HG ownership and their plant diversity could be improved by a prior typology of HGs, which could be used as a dummy variable in the models.

Three categories of HGs were distinguished in the study area: (i) herb based gardens, (ii) herb and shrub gardens and (iii) palm and lianas gardens. Herbs based gardens were the most encountered from Humid to Semi-arid zones, indicating that herbs are an invariable HG component as reported by [39, 80]. Herb and shrub based HGs were mostly encountered in Humid and Semi-arid zones while palms and lianas were mostly encountered in sub-humid and semi-arid zones. In southern Benin (Humid zone), where land availability is very low due to high population density [60], herbaceous plants are preferred over shrubs, trees and palms, presumably because of their short reproductive cycle, which allows an efficient management of land, a flexibility in species composition management as well as abundant harvests in the growing season. In middle and north Benin (sub-humid and semi-arid zones), the short reproductive cycle of herbaceous species could be a solution against the declining length of the rainy season [81]. Because of more land availability in these zones [48], HGs are larger, which allows maintenance of shrubs, palms and trees (i.e. individuals of *Parkia biglobosa, Vitellaria paradoxa* or Palm species).

According to our findings, HG structure is dynamic and it is influenced by the socio-economic conditions of the HG owner as well as ecological conditions, as reported elsewhere [49, 50]. Women, presumably because of their specific daily needs, generally owned herb-based or herbs and shrub-based HGs. These categories of HGs shelter seasonal, annual or biannual plant species that likely provide relatively stable year-round products for household consumption. Women are known to use HG products for daily consumption of the household rather than for sale or gifts [82]. In contrast, young people are mainly interested in wild or semi-wild species with attractive market values [38]. HGs owner characteristics and ecological conditions, as well as local context could also influence the structure of HGs. This has been illustrated in a recent study suggesting that market access and marketing opportunities led to changes in the structure, composition and function of HGs [40, 41].

## Conclusion

This study showed that HG ownership is more dependent upon interactions between socio-economic factors than between the individual effects of these factors. Similarly, interactions between these factors and intrinsic features of HG better explained the differences in plant diversity among HGs. While plant diversity (species richness) was not determined by age or gender of the HG owner, these variables significantly affected plant composition (prevailing plant groups) within HGs. The observed early involvement of women in home gardening evidences their traditional responsibility in being responsible for HG. Furthermore their important interest in herbs and shrubs demonstrate a gender-biased (in favor of female) asset for conservation of agro-biodiversity especially CWRs, landraces and wild leafy vegetables. Therefore, training programs aiming at agrobiodiversity conservation should focus on women.

The study also shows a positive correlation between plant diversity and HG size but a decreasing effect of owners’ age (generational) on plant diversity. In absence of interventions and in the context of current social and agricultural change, the risk of simplification and dissolution of HG, previously projected [11], seems to be a real challenge. Urgent actions are required to actively integrate HGs into national conservation strategies.

Finally we conclude that HG ownership as well as plant diversity of plant in HG are affected by many correlated factors, i.e., socio-economic, demographic, local context, ecological conditions and intrinsic features of gardens etc. Thus, miss-specification or simplification of complexity could lead to wrong models and misinterpretations. Scientists and decision makers should therefore account for that risk when formulating policies.

## Abbreviations

PGR: Plant genetic resources
HG: Home garden
HGs: Home gardens
